# Early Lymphocyte Loss and Increased Granulocyte/Lymphocyte Ratio Predict Systemic Spread of *Streptococcus pyogenes* in a Mouse Model of Acute Skin Infection

**DOI:** 10.3389/fcimb.2018.00101

**Published:** 2018-04-12

**Authors:** Torsten G. Loof, Aaqib Sohail, Mahmoud M. Bahgat, Aravind Tallam, Haroon Arshad, Manas K. Akmatov, Marina C. Pils, Ulrike Heise, Andreas Beineke, Frank Pessler

**Affiliations:** ^1^Research Group Infection Immunology, Helmholtz Centre for Infection Research, Braunschweig, Germany; ^2^Research Group Biomarkers for Infectious Diseases, TWINCORE Centre for Clinical and Experimental Infection Research, Hannover, Germany; ^3^Helmholtz Centre for Infection Research, Braunschweig, Germany; ^4^Centre for Individualized Infection Medicine, Hannover, Germany; ^5^Mouse Pathology, Animal Experimental Unit, Helmholtz Centre for Infection Research, Braunschweig, Germany; ^6^Department of Pathology, University of Veterinary Medicine, Hannover, Germany

**Keywords:** biomarker, leukocytes, lymphopenia, sepsis, skin infection, *Streptococcus pyogenes*

## Abstract

**Background:** Group A streptococci may induce lymphopenia, but the value of lymphocyte loss as early biomarkers for systemic spread and severe infection has not been examined systematically.

**Methods:** We evaluated peripheral blood cell indices as biomarkers for severity and spread of infection in a mouse model of *Streptococcus pyogenes* skin infection, using two isolates of greatly differing virulence. Internal organs were examined histologically.

**Results:** After subcutaneous inoculation, strain AP1 disseminated rapidly to peripheral blood and internal organs, causing frank sepsis. In contrast, seeding of internal organs by 5448 was mild, this strain could not be isolated from blood, and infection remained mostly localized to skin. Histopathologic examination of liver revealed microvesicular fatty change (steatosis) in AP1 infection, and examination of spleen showed elevated apoptosis and blurring of the white pulp/red pulp border late (40 h post infection) in AP1 infection. Both strains caused profound lymphopenia, but lymphocyte loss was more rapid early in AP1 infection, and lymphocyte count at 6 h post infection was the most accurate early marker for AP1 infection (area under the receiver operator curve [AUC] = 0.93), followed by the granulocyte/lymphocyte ratio (AUC = 0.89).

**Conclusions:** The results suggest that virulence of *S. pyogenes* correlates with the degree of early lymphopenia and underscore the value of peripheral blood indices to predict severity of bacterial infections in mice. Early lymphopenia and elevated granulocyte/lymphocyte ratio merit further investigation as biomarkers for systemic spread of *S. pyogenes* skin infections in humans and, possibly, related pyogenic streptococci in humans and animals.

## Introduction

Serious bacterial infections still represent an important medical problem worldwide and the identification of sensitive and specific host biomarkers for etiology, severity and progression of bacterial infections forms a crucial basis for developing the most effective strategies for antibiotic use in humans and animals. Remarkably, there are essentially no well validated host biomarkers to predict or detect secondary systemic spread in the early stages of acute bacterial skin and skin structure infections (ABSSSI), even though secondary spread to distant sites and development of sepsis are well-known phenomena during ABSSSI of both community-acquired and post-surgical etiologies in humans and in animals of veterinary importance (Pollack et al., 2015).

*S. pyogenes* is a Gram-positive pathogen that can colonize throat and skin in humans and can cause a variety of ABSSSI, ranging from localized self-limiting infections to severe and life threatening clinical manifestations due to secondary systemic spread (sepsis) or streptococcal toxic shock syndrome (STSS) (Cunningham, 2008). Approximately 18 million cases of severe *S. pyogenes* infections are recorded annually, and this pathogen remains among the top ten mortality-causing infectious agents worldwide (Ralph and Carapetis, 2013). Considering the importance of *S. pyogenes* as a causative agent of ABSSSI with a broad spectrum of clinical presentations, it is all the more surprising that there have been essentially no prospective studies aiming to identify host biomarkers for early diagnosis, severity or progression of ABSSSI due to this pathogen. In order to identify host and pathogen features responsible for systemic spread during ABSSSI due to *S. pyogenes* we used a well-defined mouse model, which we had previously employed to identify and characterize *S. pyogenes* strains of greatly differing virulence such as strains AP1 and 5448 (Fiebig et al., 2015). Mice infected with strain AP1 developed an invasive infection leading to sepsis and death within 3 days post infection (p.i.) whereas infection with strain 5448 was limited to a local infection of skin and skin structures and survival of most animals due to the absence of sepsis (Fiebig et al., 2015).

Enumeration of peripheral blood cell populations is commonly used to follow the course of infections in humans, but has been neglected in the study of small animal models of infection. This is particularly surprising as complete blood counts can be determined easily and cost efficiently in several animal species with standardized routine equipment. We have previously demonstrated that peripheral blood leukocyte indices, notably the granulocyte/lymphocyte ratio, correlate with establishment of influenza A infection in a murine model and also predict severity of the infection in mouse strains of differential susceptibility to this pathogen (Dengler et al., 2014; Preusse et al., 2015).

The aims of the present study were, therefore, to use the above-described model of *S. pyogenes* infection (1) to perform a detailed analysis of regulation of peripheral blood indices in ABSSSI due to these two *S. pyogenes* strains of greatly differing virulence and (2) to evaluate the potential of peripheral blood indices as early sensors of systemic spread and histopathological changes during the course of the infection.

## Materials and methods

### Bacterial strains and culture conditions

The *S. pyogenes* AP1 strain (40/58) of serotype M1 was originally obtained from the collection of the World Health Organization (WHO) Collaborating Center for Reference and Research on Streptococci (Prague, Czech Republic). The 5448 strain of serotype M1 was originally isolated from a patient with necrotizing fasciitis and toxic shock syndrome (Chatellier et al., 2000). Bacteria were grown overnight in Todd-Hewitt-Broth supplemented with 0.5% yeast extract at 37°C and 5% CO_2_. For infection experiments, bacteria were harvested in mid-logarithmic phase and washed with sterile phosphate buffered saline (PBS). The bacterial concentration was adjusted to 5 × 10^8^ colony-forming units (CFU)/ml in PBS.

### Mouse infection model

Female C57BL/6J mice (8 to 12 weeks old) were purchased from Harlan (Venray, The Netherlands) and housed in a pathogen-free animal facility at the Helmholtz Centre for Infection Research (Braunschweig, Germany) under standard conditions according to institutional guidelines. All animal experiments were approved by the regulatory authorities for animal experimentation (Niedersächsisches Landesamt für Verbraucherschutz und Lebensmittelsicherheit, Oldenburg, Germany; permit 33.9-42502-04-12/1009). Mice were infected subcutaneously on the back with 5 × 10^7^ CFU in 100 μl PBS. Mice injected with 100 μl PBS alone served as controls. Mice were sacrificed by CO_2_ asphyxiation 6, 12, 24, and 40 h p.i. Several independent complete time course experiments were conducted with 2–3 animals per infection type and time point, and data were pooled as indicated in the figure legends.

### Determination of bacterial loads

After the indicated time points, mice were sacrificed by CO_2_ inhalation and blood was drawn by cardiac puncture. After incubation at room temperature for 1 h, blood samples were centrifuged in a table-top microcentrifuge (Eppendorf Deutschland, Wesseling, Germany) at 12,000 rpm for 10 min to obtain serum. Aliquots of serum samples were stored at −80°C until further use. Liver, spleen, and lungs were dissected from the animals and homogenized with a disperser (Kinematica; Lucerne, Switzerland) in 5 ml sterile PBS. Blood and organ homogenates were plated onto blood agar in 10-fold serial dilutions. Bacterial loads were determined by counting colonies after 18 h of incubation at 37°C.

### Measurement of peripheral blood indices

Fifty microliter of fresh mouse blood obtained from uninfected or *S. pyogenes* infected animals were obtained by cardiac puncture, mixed with 10 μl of 0.5 mM EDTA as anticoagulant and analyzed with the VetScan HM5 system (Abaxis, Darmstadt, Germany) according to the manufacturer's instructions. The following parameters were measured: total white blood cells (WBC), neutrophilic granulocytes (GRA), monocytes (MON), lymphocytes (LYM), erythrocytes, and thrombocytes (platelets, PLT). Granulocyte/lymphocyte ratios were computed by dividing absolute or relative granulocyte counts by absolute or relative lymphocyte counts, respectively. The hematological data of five independent experiments were pooled to result in data from 13 uninfected mice and 14–15 infected mice per time point and bacterial strain used, except for the 40 h time point, at which two AP1 infected mice had died due to high virulence of this strain, resulting in *n* = 13 in that group.

### Histological examination of organs

Mice were infected as described above and liver, spleen, and lung were dissected after the indicated time points, fixed in 4% neutrally buffered formaldehyde and embedded in paraffin according to standard procedures. Sections of 3 μm thickness were stained with hematoxylin-eosin (H&E) and evaluated by light microscopy by two examiners (MCP and AB) who were blinded to the experimental groups.

Caspase-3 (Casp3) immunohistochemistry to detect apoptotic cells was performed using a polyclonal rabbit antibody directed against cleaved Casp3 (Cell Signaling, Leiden, The Netherlands, ASP175). After blocking endogenous peroxidase and heat-mediated antigen retrieval, paraffin sections were blocked with goat serum (diluted 1:5 in PBS) and incubated with the primary antibody overnight at 4°C. Sections used as negative controls were incubated with rabbit normal serum (R4505; Sigma-Aldrich Chemie GmbH, Taufkirchen, Germany) diluted 1:2000 in PBS. Goat-anti-rabbit IgG diluted 1:200 in PBS (BA-1000; H+L, Vector Laboratories Inc., Burlingame, CA, USA) was used as secondary antibody. Positive antigen-antibody reaction was visualized using the peroxidase-conjugated avidin-biotin complex (ABC) method (PK-6100, Vector Laboratories Inc.) and 3,3′-diaminobenzidin-tetrahydrochlorid (DAB) as chromogen. Finally, sections were counterstained with Mayer's hematoxylin. The number of labeled cells in the splenic white pulp was counted manually in 10 randomly chosen high power (400x) fields.

For Casp3-B220 and Casp3-CD4 double stains, sections were deparaffinized in xylene and rehydrated in graded ethanol series. After antigen retrieval and blocking of endogenous peroxidase activity, rabbit-anti-Casp3 (Cell Signaling, ASP175) and either rat-anti-B220 (eBioscience, eBioH35-17.2) or rat-anti-CD4 (eBioscience, Clone L3T4,Ly-4) were applied for fluorescence double staining with the secondary antibodies goat-anti-rat Alexa 488 (Dianova, 112-545-003) and goat-anti-rabbit Alexa 594 (Dianova, 111-585-003). DAPI (Roche) was used for counterstaining. Slides were covered with Vecta Shield Hardset (H-1400) and visualized using a fluorescence microscope.

### Statistical analyses

All statistical analyses were carried out using GraphPad Prism 5 software (GraphPad Software Inc., USA) or SPSS (IBM Analytics, Ehningen, Germany), defining a level of *P* ≤ 0.05 as significant. The multidimensional scaling (MDS) analysis was carried out with the R package MASS (https://cran.r-project.org/web/packages/MASS/MASS.pdf. Receiver operating characteristic (ROC) analysis was used to identify the discriminatory ability of biomarker candidates and was performed using SPSS Statistics for Windows (IBM Corp., Armonk, NY).

## Results

### Differences in systemic spread of *S. pyogenes* strains AP1 and 5448

**Figure 1:**
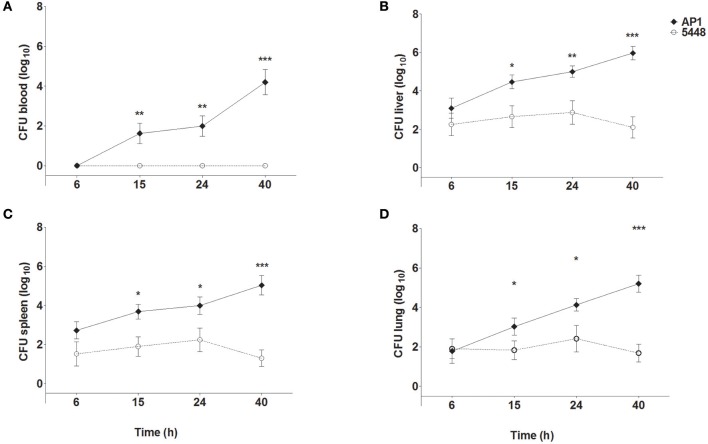
Bacterial loads in blood **(A)**, liver **(B)**, spleen **(C)**, and lung **(D)** of mice infected with *S. pyogenes* strains AP1 or 5448. Female C57BL/6J mice were infected subcutaneously on the back with 5 × 10^7^ CFU *S. pyogenes*. After the indicated time points, mice were sacrificed, blood was drawn by cardiac puncture, and livers, spleens and lungs were dissected and homogenized in PBS. Blood samples and organ homogenates were plated onto blood agar plates in 10-fold serial dilutions. Bacterial loads were determined by counting colonies after 18 h of incubation at 37°C. For simplicity sake, the value of 10^0^ (corresponding to one hypothetical colony) was inserted for all samples with sterile culture. The graphs show mean ± SD of 7–10 mice per group, combined from three independent complete time course experiments. *P ≤ 0.05, **P ≤ 0.01, ***P ≤ 0.001. Unpaired *T*-test.

Figure 1 shows bacterial loads in blood (Figure 1A), liver (Figure 1B), spleen (Figure 1C), and lungs (Figure 1D) of mice throughout the 40 h time course of infection. The more virulent AP1 strain could be detected in all three organs at the earliest time point measured (6 h p.i.), in blood starting at 15 h p.i., and bacterial loads increased steadily in all compartments through 40 h p.i. In contrast, the less virulent 5448 strain was undetectable in blood at all time points p.i. In the three organs low bacterial loads were detected, which decreased further between 24 and 40 h p.i indicating clearance of the infection. Thus, in agreement with our previous results, the AP1 strain caused a rapidly developing systemic infection (sepsis), whereas the course of infection with the 5448 strain was much less severe; notably it did not feature sepsis, but much milder internal organ involvement (Fiebig et al., 2015).

### Histopathological differences between AP1 and 5448 infection

**Figure 2:**
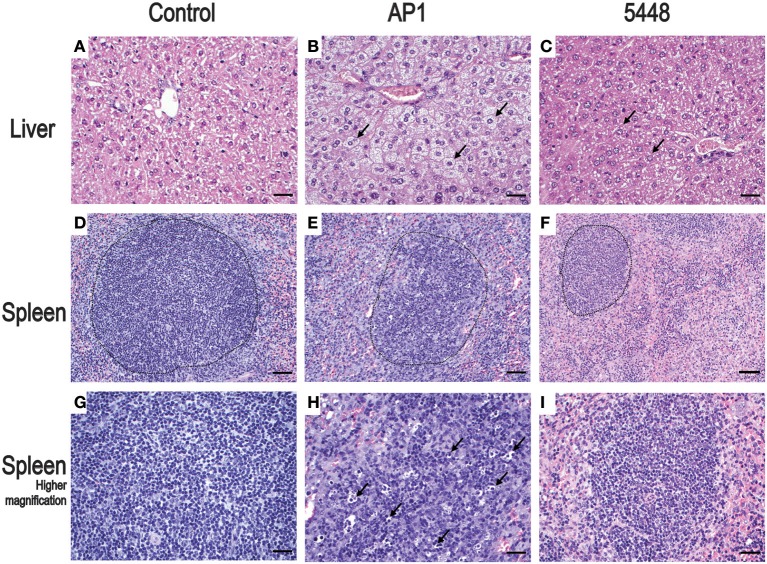
Representative H&E stained sections of the most salient changes seen in internal organs 40 h p.i. Top row **(A–C)** liver; microvesicular fatty change (intrahepatic vacuolization) in hepatocytes consistent with steatosis is seen in AP1 infection **(B)** but not in controls **(A)** or 5448 infection **(C)** (bars = 25 μm). The arrows point to selected representative hepatocytes. Middle row **(D–F)** spleen; granulocytosis and apoptosis of lymphoid cells and blurring of the border between white and red pulp in AP1 infection (compare **E** to **D** and **F**). The dotted lines delineate areas of white pulp (bars = 50 μm). Bottom row **(G–I)** higher magnification (bars = 25 μm) of white pulp. The arrows in **(H)** point to pyknotic lymphocytes amost exclusively seen in AP1 infection (compare **H** vs. **G** and **I**).

Non-infected control mice showed no pathological lesions in liver, spleen, and lung samples. The first histopathological abnormalities were seen at 24 h p.i. and generally became more severe by 40 h p.i. In lung, only an occasional hypertrophy of pneumocytes and mild pneumonitis were seen in infection with either strain. The most prominent changes at 40 h p.i. affected liver and spleen and are shown in Figure 2, compared to uninfected mice. Microvesicular fatty change (intrahepatic vacuolization), indicating steatosis, was seen in AP1-infected liver (Figure 2B). This agrees well with findings in humans, where hepatic steatosis is often observed in patients dying of septic shock (Koskinas et al., 2008). Increased numbers of neutrophilic granulocytes, as a classical sequel of bacterial infection, were found in the splenic red pulp of both groups. In addition, in AP1 infection there was some loss of normal architecture of white pulp, blurring of the borders with the transitional zone and red pulp and increased numbers of pyknotic cells (Figures 2E,H). Spleens of 5448 infected animals were smaller upon gross examination and exhibited smaller areas of white pulp, both features being consistent with emigration of lymphocytes into the periphery.

**Figure 3:**
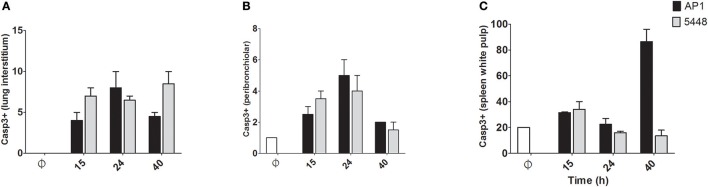
Quantitative determination of apoptosis in lung and spleen. Apoptotic cells were identified by immunostaining for cleaved Casp3. Only cells with dark staining involving the nucleus were counted as positive. In A-C the y-axis shows the average number of cleaved Casp3+ cells per 10 high-power (400x magnification) fields. **(A)** Lung (interstitium). **(B)** Lung (peribronchiolar space). **(C)** Spleen (white pulp). Graphs represent mean values obtained from the two mice per group that were used for histological examination.

**Figure 4:**
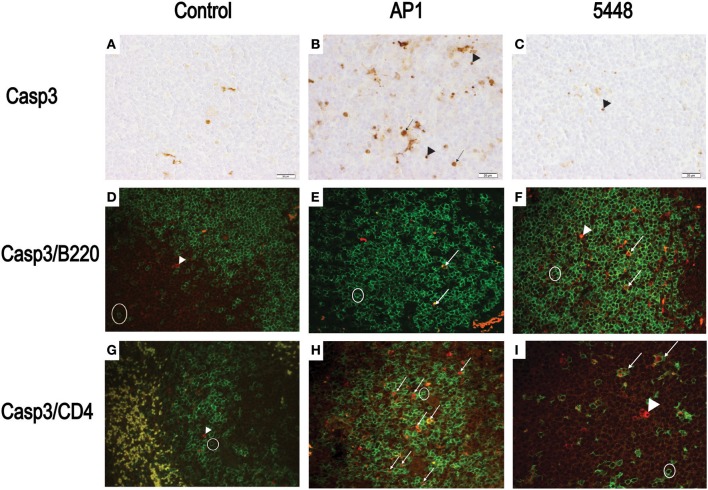
Detection of apoptotic CD4+ and B220+ cells in white pulp of spleen. **(A–C)** Representative images showing cleaved Casp3+ cells detected by immunohistochemistry. The symbols point to selected cells illustrating the detection of cleaved Casp3 in cells with lymphoid appearance (arrowheads) and in cells with morphology consistent with macrophage or dendritic cells (arrows). **(D–F)** Double immunofluorescence showing cleaved Casp3 (red) expression in B220+ (green) cells. Circle = typical B220 single positive cell; arrows = double positive cells, defined as a red nucleus within green staining membrane or yellow stain resulting from superimposition of red and green signals due to breakdown of cellular compartments late in apoptosis. **(G–I)** Double immunofluorescence showing cleaved Casp3+ (red) and CD4+ (green) cells in splenic white pulp. Circle = typical CD4+ cell; arrowhead = cleaved Casp3+ cell; arrows = double positive cells (defined as in **D–F**). Bars = 20 μm.

Quantification of apoptosis by immunohistochemical detection of cleaved Casp3+ cells showed mildly increased apoptosis in interstitial and peribronchiolar tissue in lung starting at 15 h p.i. (Figures 3A,B), with no consistent differences between AP1 and 5448 infection. In contrast, quantification of cleaved Casp3+ cells in spleen revealed a greater extent of apoptosis in white pulp, which peaked at 40 h p.i. in AP1 infection, whereas the numbers of apoptotic cells in 5448 infection were about 10-fold lower at this point (Figure 3C). This was consistent with the clinical recovery of the mice infected with this strain. Further immunohistochemical staining of spleen confirmed the white pulp as preferred site of apoptosis, predominantly affecting cells of lymphoid morphology but also large cells suggestive of being macrophages or dendritic cells (Figure 4). These apoptotic cells were markedly more frequent in AP1 infection than in control animals or 5448 infection (Figure 4, compare panel B to A and C). A markedly higher degree of CD4+ cell apoptosis was observed in AP1 infection (panel H) compared to uninfected (panel G) or 5448-infected (panel I) animals. Thus, apoptosis in spleen was markedly more pronounced in AP-1 infection and affected CD4+ cells more frequently than B220+ cells.

### Global dynamics of blood cell populations during infection

**Figure 5:**
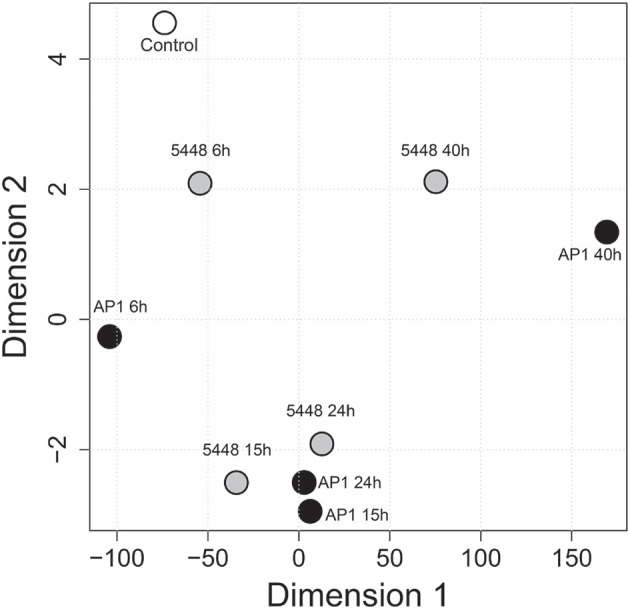
Multidimensional scaling (MDS) visualizing global across-group changes in hematologic parameters during the 40 h time course of infection. The analysis is based on median values of absolute counts of WBC, LYM, GRA, MON, PLT and the GRA/LYM ratio in peripheral blood (*n* = 13–15 mice per group, using data combined from five independent complete time course experiments featuring three mice per treatment), obtained with a VetScan hematology analyzer as described in Methods. Each circle represents the centroid of the values of the respective groups of mice. The numbers indicate the time after infection (h). AP1 infection: black fill; 5448 infection: gray fill; uninfected controls: white fill.

MDS was then used to visualize the extent of reprogramming of the hematological indices throughout the infection (Figure 5). Overall, hematological indices differed between infected and control groups at all time points p.i., and there was a temporal progression in hematological reprogramming along the time course if viewed in a counterclockwise direction. Of note, compared to 5448 infection, AP1 6 h and 40 h p.i. were clearly more distant from the values from the uninfected mice. This suggested a more rapid evolution of changes in hematological parameters in AP1 infection, but a tendency to overall normalization at 40 h p.i in 5448-infected mice, consistent with resolution of the infection and clinical recovery.

### Differences in specific blood cell lineages between AP1 and 5448 infection

**Figure 6:**
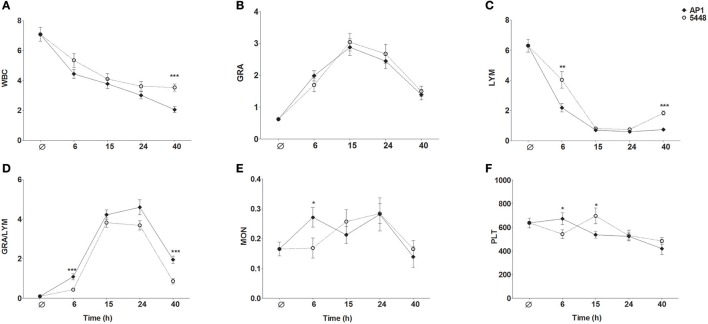
Changes in peripheral blood indices throughout the 40 h time course of infection. The same data were used as for the MDS shown in Figure 5. The graphs represent mean ± SD of 13–15 mice per group; data were combined from five independent complete time course experiments featuring three mice per treatment. *P ≤ 0.05, **P ≤ 0.01, ***P ≤ 0.001, Mann-Whitney *U*-test. The data represent absolute numbers of cells x10^9^/μl except GRA/LYM ratio, which represents the absolute granulocyte count divided by the absolute lymphocyte **(A)** WBC, white blood cell, i.e., leukocyte, counts; **(B)** GRA, granulocytes; **(C)** LYM, lymphocytes; **(D)** GRA/LYM, granulocyte/lymphocyte ratio; **(E)** MON, monocytes; **(F)** PLT, platelets (thrombocytes).

WBC count decreased steadily throughout infection with the AP1 strain (Figure 6A). During infection with the 5448 strain, there was a parallel decline in WBC count through 15 h p.i., but it stabilized subsequently. Granulocytosis peaked at 15 h p.i. in infection with either strain (Figure 6B). During infection with either strain, a marked drop in lymphocyte count was observed, but it was much more pronounced at 6 h p.i. in AP1 infection and began to normalize 40 h p.i. in 5448 infection only (Figure 6C), consistent with the beginning clinical improvement of mice infected with this strain. Of note, across all time points 55 of the 56 (98%) AP1-infected mice had elevated granulocyte counts, but lymphocyte counts were decreased in all mice. In 5448 infection, all 55 mice with granulocytosis (55/59, 93%) had decreased lymphocyte counts. Thus, granulocytosis and lymphopenia were closely associated with each other in infection with either strain. The GRA/LYM ratio increased dramatically and peaked at 15 h p.i. in 5448 infection and at 24 h p.i. in AP1-infection (Figure 6D). At early (6 h p.i.) and late (40 h p.i.) time points it was significantly greater in AP1 infection (*P* ≤ 0.001). Monocyte counts rose early in AP1 infection, but at the subsequent time points there was no significant difference between the two strains, and monocyte counts remained relatively stable throughout infection with either strain (Figure 6E). When expressing the abundances of the various leukocyte types as relative values, i.e., percentages of WBC count, the same tendencies were observed, but the differences in lymphocyte and granulocyte percentages between AP1 and 5448 infection at 6 h p.i. and 40 h p.i. were even more pronounced (data not shown due to space limitation). Platelet counts decreased gradually throughout infection with either strain (Figure 6F). Taken together, these results identified the faster evolution of lymphopenia and the absence of any sign of its reversal as the cardinal hematological features of AP1 infection in this model of ABSSSI-associated sepsis.

### Quantitative biomarker evaluation of peripheral blood cell indices

**Figure 7:**
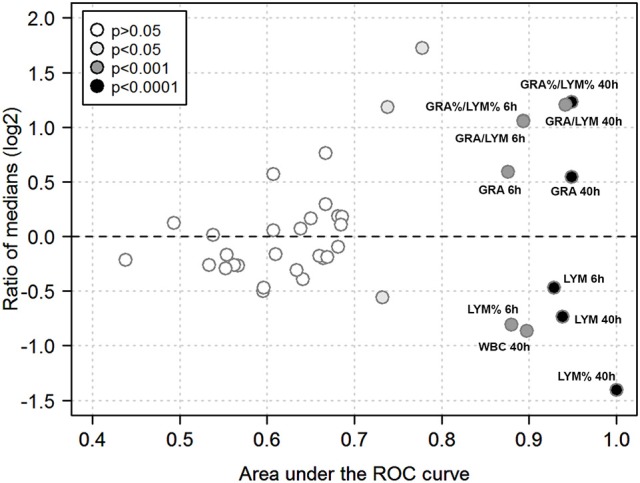
Quantitative biomarker evaluation of peripheral blood cell indices. The analysis is based on the data used for Figures 5, 6 using absolute and relative values of the hematological indices outlined in Methods. Each symbol corresponds to one blood cell parameter and time point. Ratios of medians of all paired group comparisons (“fold change” of a given parameter in AP1 infection / 5448 infection at a given time point) are plotted along the y-axis (log_2_-scale), the area under the ROC curve along the x-axis, and the asymptotic significance of the ROC curve (significance cut-off = *P* ≤ 0.05) as the fill color. Blood cell parameters of particular interest are identified by labels. Same abbreviations as in Figures 6 except that a % denotes use of a relative cell count, i.e., the absolute count of the respective cell type divided by WBC count.

We then used ROC analysis to assess the ability of the various hematological parameters to discriminate between infection with the highly virulent AP1 strain vs. the less virulent 5448 strain at any of the time points studied. To this end, areas under the ROC curves (AUC) and ratios of medians (“fold change”) were plotted against each other to visualize the most discriminatory blood cell markers (Figure 7). Several highly discriminatory markers were identified, and mostly stemmed from early (6 h) and late (40 h) time points. Since clinically relevant markers would have to be valuable at early times of infection, we focused on the 6 h time point. Here, the markedly decreased absolute LYM counts in AP1 infection (AUC = 0.93) and the GRA/LYM ratio based on absolute counts (AUC = 0.89) constituted the most highly discriminatory marker. Thus, the stronger degree of lymphocyte depletion alone constituted the most accurate prognostic/diagnostic early biomarker for systemic spread of *S. pyogenes* infection.

## Discussion

In the present work we assessed the potential of hematological parameters as biomarkers for systemic spread in a mouse model of ABSSSI with two *S. pyogenes* strains of greatly differing virulence. We identified LYM and GRA/LYM at 6 h p.i. as highly accurate early biomarkers for detection of invasive infection.

### Potential mechanisms of the strain-specific differences

The AP1 strain caused a markedly more pronounced rapid lymphocyte loss. In terms of the mechanism causing this lymphopenia, it is plausible that it is mediated by toxins expressed by group A streptococci, as it has been known for decades that streptococcal toxins can produce this phenomenon (e.g., Hríbalová et al., 1980). However, comparison of the genomic sequences of the AP1 and 5448 strains did not reveal any obvious differences in the presence of genes encoding major streptococcus-associated toxins. The AP1 strain differs from the 5448 strain in at least four major features: (1) destruction of a type II CRISPR-Cas system by phage insertion, (2) absence of the MGAS5005-like clonal complex, (3) an additional protein-H encoding gene, and (4) a mutation in the regulator gene *rofA* (Fiebig et al., 2015). It will now be important to identify the relative contributions of these genomic differences to the marked differences in virulence between the two strains. The early lymphopenia at 6 h p.i. is clearly not a consequence of the extensive apoptosis observed in spleen at the late time points (which is seen in infection with a variety of pathogens) but may be more likely due to destruction of lymphocytes in peripheral blood or, possibly, in lymph nodes, or due to sequestration at sites of infection.

### LYM and GRA/LYM ratio in human streptococcal infections

In human infection, both parameters have been studied particularly for the evaluation of sepsis risk and outcome. For instance, lymphopenia has been documented as a risk factor for poor clinical outcome in group A streptococcal infections (Megged et al., 2006), but its prognostic value early in infection has not been assessed quantitatively. To our knowledge, there are no prospective studies evaluating lymphopenia or elevated GRA/LYM ratios in the context of ABSSSI, or other initially localized infections due to *S. pyogenes* infection, which assessed systemic spread/sepsis as a study endpoint. One retrospective study from 1993 reported lymphopenia in 11 of 14 patients with severe group A streptococcal disease who had normal or elevated blood counts (Barnham and Holm, 1997), but a formal evaluation of the GRA/LYM ratio was not done. Likewise, lymphopenia is a well-documented feature of necrotizing fasciitis but the predictive value of the degree or rate of lymphocyte loss for secondary sepsis in this or other *S. pyogenes*-associated ABSSSI remains unknown (Hassell et al., 2004). Our results from this reductionist murine model suggest the need for well-powered prospective studies, particularly sampling early time points, to assess the value of these simple, easily obtained hematological markers as early prognostic biomarkers for systemic spread of *S. pyogenes* from skin and skin structures, but likely also from other body sites. Considering (1) that it has been associated with the presence of bacteremia and the outcome of sepsis independent of the pathogen (de Jager et al., 2010; Hwang et al., 2017) and (2) that we and others have identified them as markers of disease progression in murine influenza A virus infection (Dengler et al., 2014; Preusse et al., 2015), decreased LYM or elevated the GRA/LYM ratio can clearly not be considered pathogen-specific markers and would need to be used in combination with microbial diagnostics. They should be particularly useful in resource-poor health care settings where access to more sophisticated (and costly) laboratory tests is limited or in settings where a diagnosis of *S. pyogenes* is already highly suspected or proven.

### Potential value of LYM and GRA/LYM ratio in animal infections

Our results underscore the value of simple hematological parameters to detect progression of an experimental infection in a small animal model. Considering that the small volume required to obtain a complete blood count is compatible with survival of the animal, it may prove useful in scenarios where it is important to determine the extent of an infection early on. For instance, it is conceivable that hematological data, including LYM and the GRA/LYM ratio could serve as easily obtainable early endpoints in small animal studies in the context of preclinical antibiotic development. More formal assessments of lymphopenia and the GRA/LYM ratio also merit further investigation during streptococcal infections in animals of veterinary importance. *S. canis*, closely related to *S. pyogenes*, causes skin infection in dogs (Lamm et al., 2010). Here—similar to *S. pyogenes* in humans—necrotizing fasciitis can lead to secondary septicemia and toxic-shock-like syndrome, respectively (Lamm et al., 2010; Lefébure et al., 2012). Moreover, in agreement with the present findings, systemic pyogenic streptococcal infection of dogs can cause widespread lympholysis and depletion in peripheral lymphoid organs (Byun et al., 2009). However, the prognostic value of lymphopenia or an elevated GRA/LYM ratio remains to be formally investigated in this and other animal hosts.

### Further characterization of the mouse model and *S. pyogenes* strains used

The present data also provide further validation of the use of this mouse model of ABSSSI (Fiebig et al., 2015) to study the pathogenicity of and host response to *S. pyogenes* strains of differential virulence following subcutaneous inoculation. A fulminant sepsis developed in AP1 infection, but bacteremia was absent in 5448 infection. Notably, the histopathological analyses, which were not done in the original report (Fiebig et al., 2015), are consistent with the bacteriological findings that some low-grade spread to internal organs occurs even in 5448 infection, but they also clearly demonstrate that significant internal organ pathology, particularly in liver and spleen, is unique to AP1 infection.

### Outlook

Taken together, the data underscore the need for further research into mechanisms and consequences of lypmphocyte loss in *S. pyogenes* infection and strongly suggest the need for well-powered prospective studies in humans on the value of lymphocyte loss and elevated GRA/LYM ratios as early biomarkers for systemic spread of *S. pyogenes* and related pathogens during ABSSSI and, likely, infections at other body sites.

## Author contributions

FP, TL, AS, MB, AB, and MP conceived and designed the study; TL, and AS performed the experiments; AS, MA, MB, MP, and AB analyzed the data. All authors participated in writing the manuscript. FP wrote the final version of the manuscript and takes responsibility for the integrity of the data. All authors have read and approved the final manuscript.

### Conflict of interest statement

The authors declare that the research was conducted in the absence of any commercial or financial relationships that could be construed as a potential conflict of interest.
